# Feeding ecology of the common wood pigeon (*Columba palumbus*) in a major European city

**DOI:** 10.1098/rsos.231721

**Published:** 2024-07-03

**Authors:** Álvaro Luna, Fernando Pomeda-Gutiérrez, Javier Galán Díaz

**Affiliations:** ^1^ Department of Biosciences, Faculty of Biomedical and Health Sciences, Universidad Europea de Madrid, Madrid 28670, Spain; ^2^ Department of Biodiversity and Conservation, Real Jardín Botánico de Madrid (RJB-CSIC), Madrid 28014, Spain; ^3^ Department of Pharmacology, Pharmacognosy and Botany, Faculty of Pharmacy, Universidad Complutense de Madrid, Madrid 28040, Spain; ^4^ Department of Plant Biology and Ecology, Faculty of Biology, Universidad de Sevilla, Sevilla 41012, Spain

**Keywords:** urban parks, street trees, birds, exotic trees, feeding behaviour

## Abstract

Urban configuration and food availability influence birds’ foraging behaviour and constitute key factors for understanding how they exploit cities. Here, we conducted a field survey in the city of Madrid (Spain) from winter 2021 to autumn 2022 to understand how the common wood pigeon (*Columba palumbus*) exploits the food resources provided by urban parks and streets across different seasons. The proportion of observations away from parks increased during winter and spring, and the proportion of observations of wood pigeons eating on the ground was the greatest in summer. The common wood pigeon fed from 45 tree species, 60% of which were exotic ornamental species. Most tree species used as food sources coincided with those widely planted in parks, streets and avenues. The preferred trees varied throughout the year, with a greater incidence of exotic species in winter and spring. Our results show that the diversity of trees available in cities and the use of non-native plants with contrasting phenological patterns compared with the local flora are crucial elements in explaining the successful establishment of the common wood pigeon in the city.

## 1. Introduction

Urbanization threatens global biodiversity by altering natural ecosystems [1,2], usually leading towards impoverishment and homogenization of urban biological communities [3]. Still, certain species take advantage of the opportunities provided by urban habitats, increasing their demographic parameters in comparison with their rural and natural populations [4,5]. This is the case of the common wood pigeon (*Columba palumbus* Linnaeus, 1758), a granivorous–frugivorous species traditionally restricted to woodlands and cultivated fields [6–8], and whose population has increased over the last three centuries across its distribution range including a greater presence in urban areas [9–11]. We argue that understanding the relationship between the foraging behaviour of native birds and the distribution of green features in the urban landscape is a powerful approach to promote biodiversity in cities.

The structure of urban landscapes influences the composition of bird communities and how these exploit urban environments [12,13]. For instance, there is a positive relationship between the cover and diversity of urban green spaces and the richness, diversity and abundance of native birds [14–18]. Wooded streets also play a key role for bird communities within cities because they can be used as a food resource outside parks [19] and act as corridors that connect parks and peri-urban areas [20,21]. Ornamental trees, in particular, are an important element for the establishment of urban animal populations, mainly by increasing the total plant diversity in cities and the spatial and temporal offer of resources [22–24]. Frugivorous birds benefit from the presence of ornamental species because most of them have edible fruits and seeds [25–27].

The first records of urban wood pigeons breeding in European cities were reported in western and central Europe in the early nineteenth century [28,29]. Subsequently, they colonized northern European cities (from Denmark to Finland) during the twentieth and twenty-first centuries [30]. Currently, the northeastern limit of the progressive urban colonization of wood pigeons coincides with Lithuania, Belarus and Ukraine [9]. In Spain, the first records of urban common wood pigeons were registered in the last decades of the twentieth century in Madrid [31], and recently, the species has started to colonize other Spanish urban and peri-urban areas where they establish growing populations with higher densities than in other habitats [11].

Previous studies have explored the diet of the common wood pigeon in Mediterranean areas. Gutiérrez-Galán *et al.* showed how the diet of the common wood pigeon in southern Spain varies during the year: while the most consumed item during winter are acorns of the genus *Quercus*, during summer their first choice is cereals, and in spring and autumn, their diet is dominated by tree fruits [32]. Similarly, Kaouachi *et al.* confirmed a seasonal diet pattern of common wood pigeons in a Mediterranean forest in Algeria: drupes of *Pistacia lentiscus* L. and acorns of *Quercus suber* L. dominated the diet in autumn and winter, and a greater consumption of olive fruits and *Pinus halepensis* Mill. seeds was found in spring [33]. The common wood pigeon tends to occupy parks and less disturbed areas of the city [21,27]. However, little is known about the foraging behaviour of the common wood pigeon in cities, and the relative importance of ornamental and native plants in their urban diet, despite both resources being known to be exploited by this species [11]. In this regard, we argue that the high plasticity in feeding habits shown by common wood pigeons allows them to exploit and benefit from the wide range of resources available from trees in cities.

In the present study, we surveyed common wood pigeons in one of the largest European cities for an entire year using direct observations. Our main hypothesis was that the diversity and distribution of greenery in cities is key to understanding the foraging behaviour of frugivorous/folivorous/granivorous birds such as common wood pigeons. Our objectives were to (i) analyse the differential use of parks, wooded streets and avenues by common wood pigeons throughout the year, and whether they obtain their resources from trees or the ground; (ii) examine how the tree-based diet of common wood pigeons varies across seasons, by analysing the most frequently consumed taxa and the organs they feed on (roots, leaves, fruits and seeds); and (iii) explore the relative importance of ornamental species in the diet of common wood pigeons, with a special focus on when and to what extent they use exotic species as a food resource.

## 2. Material and methods

### 2.1. Study area

The study area is located within the municipality of Madrid, the capital and most populated city of Spain (electronic supplementary material, appendix S1). The municipality of Madrid is located in the centre of the Iberian Peninsula (40.42° N, 3.70° W). It has a Mediterranean climate with hot summers and cold winters with precipitations mostly concentrated in spring and autumn (Csa Köppen-Geiger climatic classification) [34].

The municipality of Madrid has around 481 000 trees which mostly belong to 50 species, of which 62% are exotic [35]. Madrid parks host a great diversity of species, e.g. 336 tree species grow in Real Jardín Botánico de Madrid (Real Jardín Botánico; rjb.csic.es 2023), 167 in Parque de El Retiro (Ayuntamiento de Madrid 2014) and 117 in Parque del Oeste [36]. The most abundant angiosperm species in Madrid’s streets and parks are *Platanus* × *hybrida* Brot. (94 724 individuals), *Ulmus pumila* L. (51 108 individuals), *Styphnolobium japonicum* (L.) Schott (43 318 individuals) and *Celtis australis* L. (39 323 individuals) [35]. These, along with *Aesculus hippocastanum* L, and species from the genera *Morus* and *Ligustrum* constitute the most conspicuous species.

### 2.2. Data collection

From October 2021 to September 2022, two people surveyed the feeding patterns of the common wood pigeon. To detect common wood pigeons eating both on the ground and trees, we walked transects in parks and streets (henceforth ‘urban matrix’). Urban matrix refers to streets, avenues, small parks (less than 5 ha) and public and private gardens integrated into the study area. ‘Parks’ refer to the historical green areas located within the city: Parque de El Retiro (114.19 ha), Parque del Oeste (79 ha), Parque de la Dehesa de la Villa (64.17 ha), Real Jardín Botánico de Madrid (7.8 ha) and Quinta Fuente del Berro (7.42 ha) [35] (electronic supplementary material, appendix S2); and other large green areas such as Campo del Moro and Parque de Atenas (which together form a continuum of 274 ha) and 6.5 km of the urban section of the Manzanares river together with its adjacent parks, especially Parque Madrid Río (approximately 35 ha). Moreover, we classified the observations as ‘ground’, when the pigeons were observed feeding on grass, seeds and small stones on the ground, and ‘trees’ when we observed them feeding from branches of trees. When several wood pigeons were spotted on the same tree, they frequently exploited the same resource. Thus, when several individuals of the common wood pigeon were observed on the same tree, we registered the abundance. We classified the trees as native or exotic according to Flora Ibérica [37]. In the case of uncertainty in the identification of the tree species, we consulted the inventory of trees published for each park [35] and used the mobile phone applications Pl@ntNet [38] and ‘Un alcorque, un árbol’ [39].

We walked a total of 307 transects alternating morning/afternoon: 141 in parks and 166 in the urban matrix. This is an average of 15 transects per month and 45 times per season. Here, the term season follows the traditional definition for the Mediterranean region: spring (March to June), summer (June to September), autumn (September to December) and winter (December to March). In summer, late spring and early autumn, the census was conducted between 3 hours after sunrise and 3 hours before sunset. During the coldest periods of the year, especially in winter when the days are shorter, the census was conducted from 10.00 to 12.00 and 16.00 to 18.00. In parks and gardens, we covered the available pedestrian trails. In the urban matrix, we covered as many streets as possible in each transect, without passing through the same street twice and avoiding those that had previously been discarded for lacking trees. We used binoculars to avoid misidentification with other birds and to be sure of the items consumed by the pigeons in each tree. We included only those observations in which the pigeons were seen feeding, being conservative if we had doubts about what part of the tree they were eating. We excluded non-feeding events: drinking, sunbathing, resting, collecting nest material or moving among the branches without clearly perceiving any foraging behaviour. Data are publicly available at Zenodo [40].

### 2.3. Data analysis

First, we investigated the use of parks/urban matrix and trees/ground by common wood pigeons per season. We used chi-squared tests to test whether the observed patterns within seasons were significant. The Spanish Information System on Land Occupation (SIOSE) was used as the base map for representation [41]. Second, we explored changes in the relative proportion of observations of common wood pigeons per tree species and month over the study period using a locally estimated scatterplot smoothing (LOESS). We tried several smoothing parameters (α) and retained 0.75 because it captured well differences between months. Third, we explored which organs (shoots, seeds/fruits, flowers and roots) were consumed by wood pigeons, and whether they got them from native or exotic tree species. We grouped tree species at the genus level to avoid misidentifications, under the assumption that common wood pigeons equally feed on the resources provided by congeneric tree species. Fourth, we explored the spatial and temporal complementarity of tree resources used by common wood pigeons in Madrid. For this, we used the tree inventory from the Parks and Gardens Service of Madrid City Council [35] and pollen concentration provided by Red PALINOCAM [42]. To reflect floral phenology, pollen concentration data were transformed into a pollen calendar using the R package AeRobiology [43].

All analyses were performed in R (v. 4.3.1) [44].

## 3. Results

**Figure 1.**
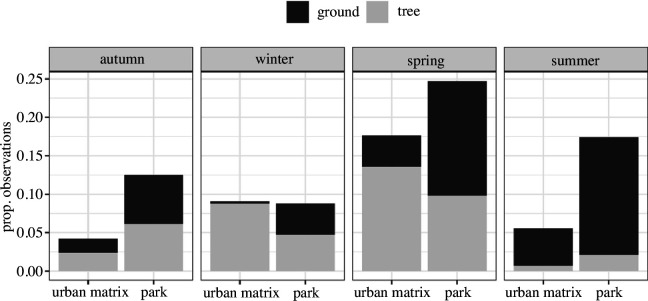
Proportion of total observations of common wood pigeons in Madrid feeding in parks and the urban matrix, and from trees and ground, grouped by season (*n* = 2922). Period: October 2021 to September 2022. Seasons: spring (March to June), summer (June to September), autumn (September to December) and winter (December to March).

**Figure 2.**
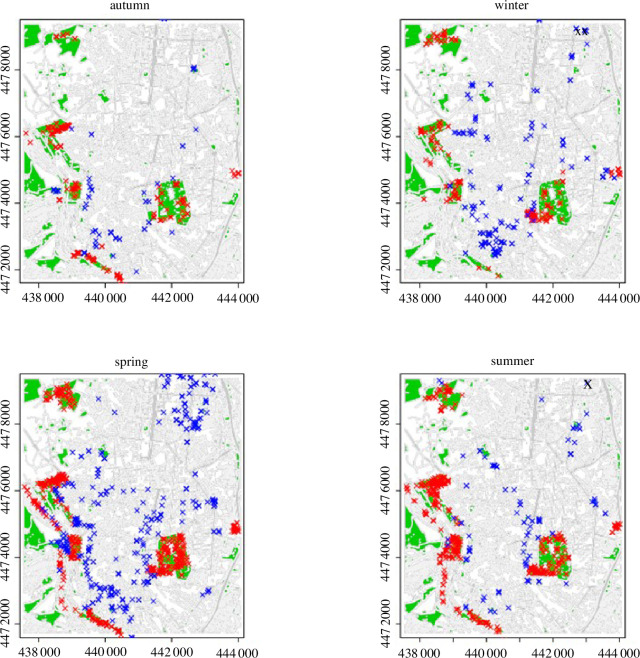
Observations of common wood pigeons in the urban matrix (blue) versus parks (red) in Madrid grouped per season. Parks are represented in green. Period: October 2021–September 2022. Seasons: spring (March to June), summer (June to September), autumn (September to December) and winter (December to March).

We recorded 2922 observations of common wood pigeons in the city of Madrid from October 2021 to September 2022 (electronic supplementary material, appendix S1). We registered 63.44% of observations in parks (36.56% in the urban matrix) and 51.77% on the ground (48.23% on trees). Common wood pigeons preferentially exploited parks across the year except in winter, when they were found feeding equally frequently in the urban matrix and parks (figure 1). During summer, we observed a clear trend towards feeding on the ground both in parks and the urban matrix, with trees having an incidental role in their diet (figure 1). In autumn, common wood pigeons found in parks and the urban matrix fed similarly on the ground and trees. In winter and spring, wood pigeons found on the urban matrix tended to feed most frequently from trees, whereas common wood pigeons found in parks fed similarly on ground and trees (winter: *X*^2^(1, *n* = 523) = 123.39, *p* < 0.05; spring: *X*^2^(1, *n* = 1238) = 166.82, *p* < 0.05). The location of observations can be seen in figure 2.

Common wood pigeons fed on 45 tree species (31 genera) during the study period, of which 26 were exotic species. Spring was the season when wood pigeons fed on the greatest diversity of trees (28 species) followed by winter (22 species), autumn (15 species) and summer (8 species). Five tree species that accumulated the most observations during the study period were *Platanus* × *hybrida* Brot. (16.25% of total observations), *Ulmus pumila* L. (15.40%), *Styphnolobium japonicum* (L.) Schott (13.83%), *Celtis australis* L. (10%) and *Morus alba* L. (8.58%) (electronic supplementary material, appendix S3). We show the results of tree genus usage by season in table 1. The 12 tree genera most used by common wood pigeons in the study area are widely planted across all historical parks (electronic supplementary material, appendix S2).

**Table 1.** Number of observations of common wood pigeons feeding from trees in the city of Madrid during the study period (October 2021–September 2022) grouped by genus and season (*n* = 1409). Observations from parks are in regular font and those from the urban matrix are in bold. Seasons: spring (March to June), summer (June to September), autumn (September to December), winter (December to March).

Genus	autumn	winter	spring	summer
*Acer*	0/**0**	6/**8**	5/**6**	0/**2**
*Aesculus*	0/**0**	1/**0**	27/**26**	0/**0**
*Arbutus*	8/**1**	0/**0**	0/**0**	0/**0**
*Carpinus*	0/**0**	1/**0**	1/**0**	0/**0**
*Catalpa*	0/**0**	0/**0**	2/**0**	0/**0**
*Celtis*	95/**6**	37/**3**	1/**1**	12/**2**
*Cornus*	0/**0**	0/**0**	2/**0**	0/**0**
*Crataegus*	4/**0**	0/**0**	0/**0**	0/**0**
*Fraxinus*	1/**0**	0/**0**	13/**0**	0/**0**
*Gleditsia*	0/**0**	0/**3**	2/**2**	0/**0**
*Gymnocladus*	0/**0**	1/**0**	0/**0**	0/**0**
*Ilex*	0/**1**	0/**0**	0/**0**	0/**0**
*Ligustrum*	16/**12**	24/**27**	0/**1**	0/**0**
*Liquidambar*	0/**1**	0/**0**	0/**0**	0/**0**
*Maclura*	0/**0**	1/**0**	0/**0**	0/**0**
*Melia*	0/**0**	0/**52**	6/**0**	0/**0**
*Morus*	0/**0**	0/**0**	78/**15**	34/**1**
*Olea*	8/**4**	0/**2**	0/**0**	1/**8**
*Photinia*	0/**0**	0/**0**	5/**0**	0/**0**
*Platanus*	0/**25**	13/**22**	64/**105**	0/**0**
*Populus*	8/**0**	0/**1**	12/**2**	0/**0**
*Prunus*	0/**0**	6/**9**	6/**11**	0/**0**
*Quercus*	32/**2**	6/**0**	1/**0**	0/**0**
*Robinia*	0/**0**	0/**0**	29/**36**	0/**0**
*Salix*	0/**0**	0/**0**	8/**1**	0/**0**
*Sambucus*	0/**0**	0/**0**	0/**0**	3/**0**
*Styphnolobium*	5/**8**	32/**89**	4/**39**	11/**7**
*Taxus*	2/**0**	0/**0**	0/**0**	0/**0**
*Tilia*	0/**9**	2/**0**	0/**0**	0/**0**
*Ulmus*	0/**0**	9/**40**	21/**152**	0/**0**
*Viburnum*	0/**0**	0/**0**	0/**0**	1/**0**

**Figure 3.**
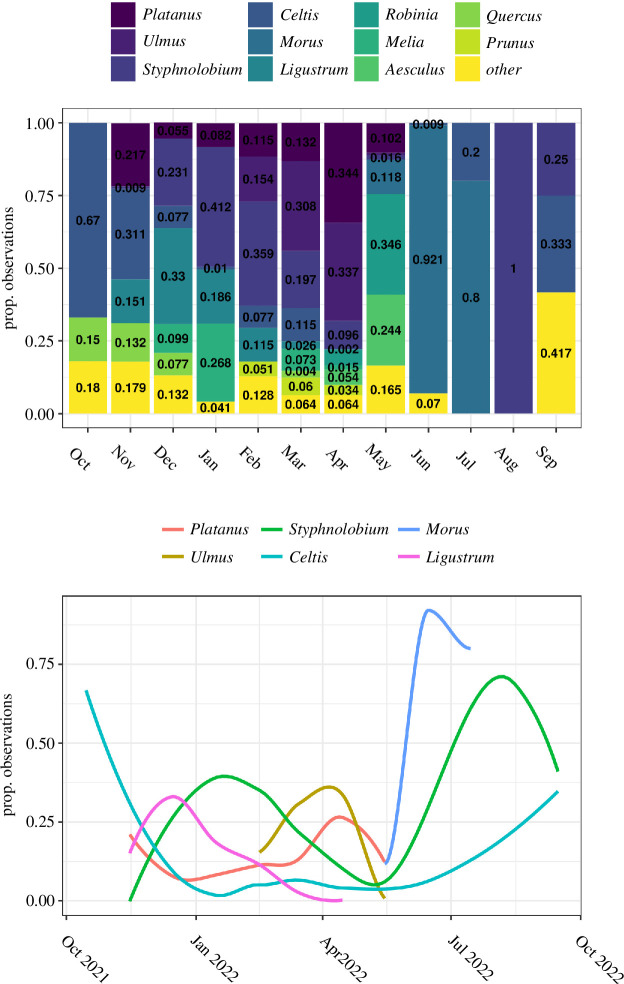
Top: proportion of observations of common wood pigeons eating from trees per month. Bottom: shifts in urban tree genera preference shown by common wood pigeons in Madrid estimated using a locally estimated scatterplot smoothing (LOESS) with the smoothing parameter (*α*) set to 0.75. Period: October 2021–September 2022.

**Figure 4.**
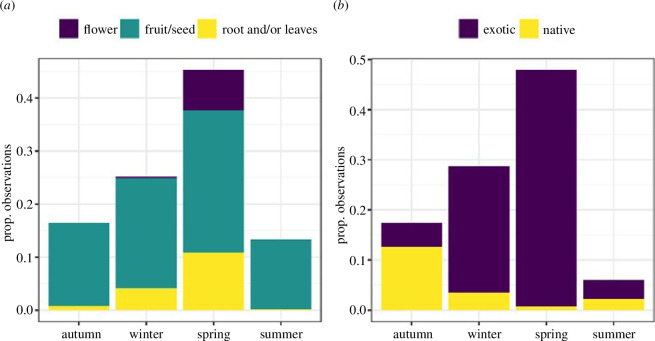
(*a*) diet of common wood pigeons in Madrid grouped by season. (*b*) tree preference (exotic/native) shown by common wood pigeons in Madrid grouped by season. Period: October 2021−September 2022. Seasons: spring (March to June), summer (June to September), autumn (September to December) and winter (December to March).

The temporal foraging patterns of the common wood pigeon varied across the year (figure 3) and coincided to a greater extent with the floral phenology of the native and ornamental trees found in the city (electronic supplementary material, appendix S4). During autumn, common wood pigeons were observed feeding mostly on the mature fruits of species from the genus *Celtis*, making up 40.24% of the observations of this season. As winter arrived, the availability of exotic *Ligustrum* sp. and *Styphnolobium japonicum* (L.) Schott fruits increased, hence accumulating most of the observations of consumption during this period (figure 3). The exotic species *Styphnolobium japonicum* (L.) Schott dominated the diet of the species throughout winter, accumulating 29.12% of the observations of this season. *Ulmus* sp. flowers are fully open in late winter, and fruits start to develop shortly after. Towards the end of winter and the beginning of spring, common wood pigeons frequently consumed seeds, and to a lesser extent leaves and shoots (figure 4), mostly of *Ulmus* sp. and *Platanus* sp. (seeds of smaller size in the case of *Platanus* sp.). *Ulmus* sp. and *Platanus* sp. constituted 21.93% and 21.42%, respectively, of the diet of wood pigeons in spring. The consumption of fruits of *Styphnolobium japonicum* (L.) Schott ceased to be preferred after winter, but as some fruits remained hanging on the trees, the pigeons continued to feed on them making it the third most used tree in spring (12.93% of the observations in this season). Also, the consumption of flowers becomes noticeable in spring (figure 4), mostly of *Aesculus hippocastanum* L. although it is not included among the most consumed plants. The preferential consumption of these species dropped abruptly as spring progressed and, towards the end of this season, the tree genus in which most common wood pigeons were observed feeding is *Morus* sp. (11.79% of the observations in spring), sometimes accumulating a great abundance of pigeons feeding on their ripe fruits. During the summer, the importance of the trees in the diet decreased and the species began to feed preferentially on the ground.

## 4. Discussion

We have analysed the seasonal and spatial foraging patterns of the common wood pigeon across one year in one of the largest metropolitan areas in Europe, basing our study on direct observations of the consumption of roots, leaves, fruits, seeds and flowers. To the best of our knowledge, this is the most comprehensive study on the diet of common wood pigeons in urban environments and one of the most exhaustive approximations on the differential use of the urban landscape by an urban frugivorous/folivorous/granivorous bird.

### 4.1. Foraging behaviour of common wood pigeons in a city during the year

Spring was the season when most observations of common wood pigeons were registered, probably because of the presence of migrating individuals that use the city as a stop-over during their travel to the north [9,11]. Moreover, there is an increase in the total number of individuals observed around this period of the year because the fledglings leave the nest and join the adult population [11]. Common wood pigeons might also be easier to detect in the city in spring because they find more resources available in this habitat in this period of the year, while in other seasons, they may preferentially exploit resources from peri-urban and rural areas [32].

Overall, we accounted for more records of common wood pigeons eating inside parks. This is an expected result as common wood pigeons reach higher abundances and breeding densities in green areas within cities [45] where there is lower level of disturbance by traffic or pedestrians [21,27]. We also found an overall greater proportion of observations of common wood pigeons foraging on the ground, mostly on lawns in summer (Álvaro Luna, personal observations). This may be owing to a greater availability of food on the grass in summer, either owing to the accumulation of seeds or the permanent irrigation of lawns, which maintains patches of green vegetation that contrast with the drier surrounding areas. Common wood pigeons were found eating on the ground mostly in parks, but in summer, they were also observed exploiting small patches of lawns in streets, avenues and small gardens (Álvaro Luna, personal observations during Summer 2022). Unlike feral pigeons (*Columba livia* Gmelin, 1789), common wood pigeons rarely use garbage when foraging in the urban matrix [46,47]. We did not observe any individual of common wood pigeon eating waste or food subsidized by citizens at the ground level. During our study, feral pigeons and common wood pigeons were not frequently observed interacting or feeding together, showing a low level of interspecies interaction, as previously shown by Fernández-Juricic [45]. Towards winter and spring, common wood pigeons appeared with a higher frequency outside parks, feeding mostly on trees in different parts of the urban matrix. This may be because the trees mostly used in streets and avenues offer a constant, abundant and predictable food reserve in that period of the year so that more birds come to exploit it (see the next section for further details).

### 4.2. Tree-based diet of common wood pigeons through the year

We found that common wood pigeons fed on different parts from 45 tree species across the year. Thus, our results support that a generalist forager such as the common wood pigeon shows great plasticity in its foraging behaviour and exploits a wide range of resources from city trees. This plasticity is evident from March to September, which coincides with their breeding season [11]. The most used tree taxa by wood pigeons (i.e. *Celtis australis* L., *Ligustrum* sp., *Styphnolobium japonicum* (L.) Schott, *Platanus* × *hybrida* Brot., *Ulmus* sp., etc.) coincide with those widely used across Madrid parks. Thus, there is a strong relationship between the diversity and distribution of trees used in urban planning and the establishment of urban populations of birds [19].

Our results also highlight the importance of ornamental exotic trees in the urban diet of common wood pigeons, contrasting with the predominant diet based on native plants observed in this species in less urbanized Mediterranean areas [32,33]. This coincides with the observed pattern in other urban birds that also include exotic species in their diet, which has been reported to contribute to maintaining their populations [22,48,49]. In our case, the contrasting flowering and fruiting phenology of these trees allowed the common wood pigeon to cover the demand for food along the different seasons, mainly in winter and early spring, helping to promote the exploitation of the urban resources by the common wood pigeon. For instance, *Celtis* sp., *Ligustrum* sp. and *Styphnolobium* sp. flowered in April, summer and late summer, respectively, and common wood pigeons fed on the fruits of these species as they were becoming mature.

It has been shown that urbanization consistently advances spring phenophases and extends the length of the growing season of vegetation types across Europe [50,51]. In this context, urban green areas might continue offering abundant and predictable feeding resources during the first stages of the breeding season and the migration of many bird species.

## 5. Conclusions

These results shed light on the mechanisms that allow omnivore and granivore birds to thrive at higher levels of urbanization [52–54]. Street trees and green urban areas in Madrid provide foraging opportunities for common wood pigeons throughout the year. The diversity of trees exploited, the use of non-native plants with contrasting phenological patterns compared with the local flora and the longer fruiting periods of some trees constitute key factors to explain the successful exploitation of the common wood pigeon in the city, as observed in other species inhabiting cities [22,49,55,56]. This approach allowed us to identify the consumption of organs such as roots, leaves, seeds, fruits and flowers, which are difficult to identify visually in faeces. Future research should focus on obtaining a more complete vision of the diet of this species in cities, for instance analysing droppings to obtain more information on what they eat at ground level. Similar studies in cities at other latitudes with different levels of tree diversity in their parks and streets, and comparisons of urban with peri-urban and adjacent rural areas, could offer a complementary vision to our results and contribute to a better understanding of the role of exotic plants in the diet of the common wood pigeon in urban ecosystems.

## Data Availability

The data and codes used in this study are available at Zenodo [57] and GitHub [58]. Supplementary material is available online [59].
